# Mitochondrial genome sequence of tuber-bearing wild potato, *Solanum commersonii* Dunal

**DOI:** 10.1080/23802359.2018.1437826

**Published:** 2018-02-10

**Authors:** Kwang-Soo Cho, Ji-Hong Cho, Ju-Sung Im, Jang-Gyu Choi, Young-Eun Park, Su-Young Hong, Tae-Ho Park

**Affiliations:** a Highland Agriculture Research Institute, Natioanl Institute of Crop Science, Rural Development Administration, Pyeongchang, South Korea;; b Department of Horticulture, Daegu University, Gyeongsan, South Korea

**Keywords:** Wild potato, *Solanum commersonii*, mitochondrial genome, Solanaceae

## Abstract

We report two complete mitochondrial genome sequences of a tuber-bearing wild potato species (*Solanum commersonii*). The genomes are circular DNA molecules with lengths of 213,676 bp and 338,427 bp containing 80 nonredundant genes totally, including 34 protein-coding genes, 25 hypothetical open reading frames, 18 tRNA genes, and 3 rRNA genes. Phylogenetic analysis using common protein-coding sequences confirmed that *S. commersonii* belongs to the Solanoideae subfamily in the Solanaceae family.

*Solanum commersonii* is a tuber-bearing wild potato species native to Central and South America (Hawkes 1990). Because of its differences in several desirable characteristics from the cultivated potato (*S. tuberosum*) (Spooner et al. 1991; Rodriguez and Spooner 2009; Cho et al. 2016), the species has been used for potato breeding (Laferriere et al. 1999; Kim-Lee et al. 2005; Cho et al. 2016). However, the two species are sexually incompatible because of different endosperm balance numbers (EBNs) and ploidy levels of the genomes, with *S. commersonii* and *S. tuberosum* reported as diploid and tetraploid with EBN values of 1 and 4, respectively (Johnston et al. 1980; Ortiz and Ehlenfeldt 1992; Cho et al. 1997). Therefore, in this study, we sequenced the entire mitochondrial genome of the wild potato, *S. commersonii* for further investigation of its evolutionary aspects. This is the first report on a wild potato species.

Total genomic DNA was isolated from fresh leaves of the *S. commersonii* breeding line (specimen deposition no. Lz3.2) and used to construct a paired-end (PE) library with an insert size of ∼670 bp, according to the standard Illumina protocol for PE library construction. The library was sequenced using an Illumina MiSeq platform by LabGenomics (www.labgenomics.co.kr, Seoul, Korea), and approximately 6.8 Gb of PE reads were obtained. After removing low-quality (Q < 30) reads using CLC quality trim (ver. 4.21, CLC Inc., Denmark), approximately 4.8 Gb of high-quality reads (approximately 91× coverage for complete mitochondrial genomes) were assembled *de novo* with CLC genome assembler (ver. 4.21), as described previously (Cho et al. 2015). The longest contigs of the mitochondrial genome sequences were selected, extended, and joined via a series of PE read mapping and gap filling. The assembled mitochondrial genome sequences were corrected manually based on read mapping status and BLASTN search. The genome sequences were annotated using GeSeq (https://chlorobox.mpimp-golm.mpg.de/geseq-app.html), and manually curated based on BLAST searches.

The two complete mitochondrial genome sequences of *S. commersonii,* which are circular DNA molecules with lengths of 213,676 bp with a G + C content of 45.5%, and 338,427 bp with a G + C content of 45.0% were assembled independently. The two genomes showed 48.3% sequence similarity, and had two collinear regions with lengths of 126 kb and 12 kb between each other. The numbers of genes predicted were 65 and 51, respectively, in the two genomes, constituting approximately 12.8% and 13.75%, respectively, the two genome sequences. Among the predicted genes, 10 protein-coding genes, 14 hypothetical open reading frames (ORFs), 2 rRNA genes, and 8 tRNA genes were commonly present between the two genomes. Overall, 80 unique genes, including 34 protein-coding genes, 25 ORFs, 18 tRNA genes, and 3 rRNA genes were identified in the genomes. The exons of the *trans*-spliced genes, *nad1, nad2*, and *nad5*, exist completely or partially in each genome.

Phylogenetic analysis was performed based on multiple alignments of common 23 protein-coding sequences in mitochondrial genome, and confirmed that *S. commersonii* belongs to the Solanoideae subfamily in the Solanaceae family (Figure 1).

**Figure 1.**
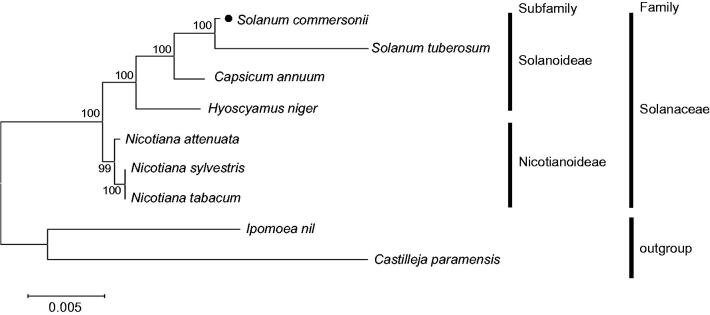
Maximum likelihood (ML) tree of nine Solanaceae-related species. The 23 protein-coding sequences in mitochondrial common gene were aligned using MAFFT (http://mafft.cbrc.jp/alignment/server/index.html). And then, phylogenetic tree was constructed with tamura-nei model and 1000 bootstrap method by MEGA 7.0 (Kumar et al. 2016). Mitochondrial genome sequences used for this tree are *Capsicum annuum*, NC_024624; *Castilleja paramensis*, NC_031806; *Hyoscyamus niger*, NC_026515; *Ipomoea nil*, NC_031158; *Nicotiana attenuate*, MF579563; *N. sylvestris*, NC_029805; *N. tabacum,* NC_006581; *S. commersonii*, MF989960 and MF989961; *S. tuberosum,* MF989953 to MF989957.
